# Supplementary material for: Lymphatic vessels interact dynamically with the hair follicle stem cell niche during skin regeneration in vivo
Source: EMBO J. 2019 Sep 2;38(19):e101688. doi: 10.15252/embj.2019101688 (PMC6769427; doi:10.15252/embj.2019101688)
Supplement: Supplementary file 1 — Appendix [file EMBJ-38-e101688-s001.pdf]

Table of Content:

Appendix Table S1. Primary antibodies.

Appendix Table S2. Secondary antibodies.

Appendix Table S3. List of RT-qPCR primers.

Appendix Table S1. Primary antibodies.

Antibody	Reference	Company
AE15	Ab58755	Abcam
BrdU	AB6326	Abcam
Cleaved caspase3	9661	Cell signaling
CD34	553731	BD Biosciences
Emilin1	103-M80	ReliaTech
GATA3	SC-268	Santa Cruz Biotechnology
JUP	CSB-PA12839A0Rb	CUSABIO
K6	PRP-169P	Covance
Ki67	MAD-000310QD	Master Diagnostica
Lhx2/9	Thomas Jessel lab	Columbia University
LYVE1	ab149117	Abcam
P-cadherin	13-2000Z	Invitrogen
Smooth Muscle Actin	MS-113-P0	ThermoFisher Scientific
Tenascin C	NB110-68136	Novus Biologicals

Appendix Table S2. Secondary antibodies.

Antibody	Reference	Company
Anti-Rabbit FITC	711-095-152	Jackson immunoresearch
Anti-Rabbit Alexa Fluor 594	711-585-152	Jackson immunoresearch
Anti-Rat Alexa Fluor 594	712-585-150	Jackson immunoresearch
Anti-Mouse Alexa Fluor 488	715-545-151	Jackson immunoresearch

Appendix Table S3. List of RT-qPCR primers.

**Primer**	**Sequence**
ACTIN RW	GTGGTGGTGAAGCTGTAGCC
ACTIN FW	GGCACCACACCTTCTACAATG
WLS FW	ATGACCTCCAATGGTGGCTG
WLS RW	CCCCTATTGGCCCAAACCAT
