# Supplementary material for: Lymphatic vessels interact dynamically with the hair follicle stem cell niche during skin regeneration in vivo
Source: EMBO J. 2019 Sep 2;38(19):e101688. doi: 10.15252/embj.2019101688 (PMC6769427; doi:10.15252/embj.2019101688)
Supplement: Supplementary file 2 — Expanded View Figures PDF [file EMBJ-38-e101688-s002.pdf]

Expanded View Figures

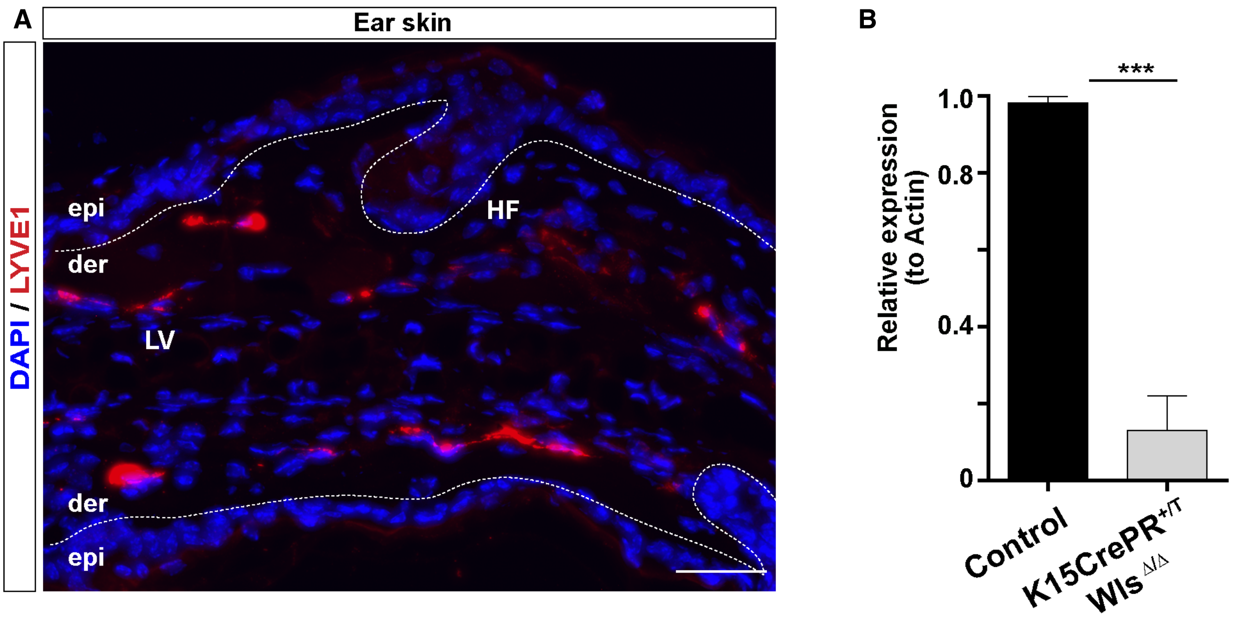

Figure EV1. Distribution of LV in the ear skin and validation of the reductions of Wls expression in the K15CrePR$^{+/T}$; Wls$^{\Delta/\Delta}$ mouse model.

A Adult ear skin sections immunostained for LYVE1 (red) and counterstained with DAPI (blue). Scale bar, 50 μm. LV, lymphatic vessels; HF, hair follicles; epi, epidermis; der, dermis.

B Histogram of the RT–qPCR analyses of the relative expression of Wls in the HFSC isolated from the K15CrePR$^{+/T}$; Wls$^{\Delta/\Delta}$ mouse model and controls. n = 3–4 skin samples per mouse, n = 3–4 mice. Data represent the mean value ± SEM. ***P < 0.001 (Mann–Whitney U-test).

 © The Authors

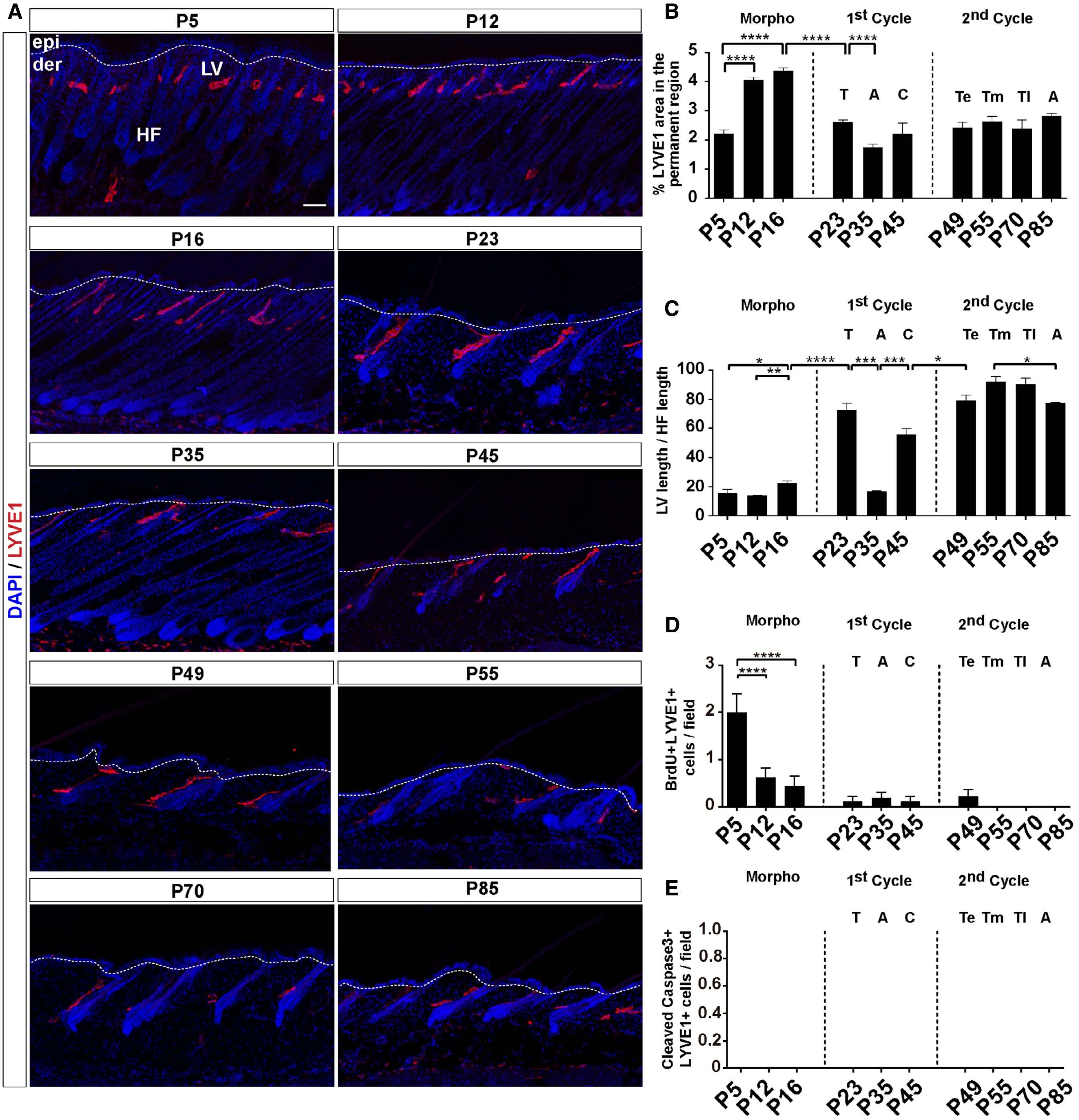

Figure EV2. LV association with HF during the postnatal HF cycle.

A Adult back skin sections from different postnatal (P) days immunostained for LYVE1 (red) and counterstained with DAPI (blue). *n* = 3–4 skin samples per mouse, *n* = 3–4 mice. Scale bar, 50 μm. epi, epidermis; der, dermis; LV, lymphatic vessels; HF, hair follicle.

B Histogram of the percentage of LYVE1-positive area in the HF permanent region at different postnatal days.

C Histogram of the percentage of LV length relative to the HF length in the back skin at different postnatal days.

D Histogram of the number of $BrdU^+$ $LYVE1^+$ cells/field in the back skin at different postnatal days.

E Histogram of the number of cleaved caspase-3^+ $LYVE1^+$ cells/field in the back skin at different postnatal days.

Data information: The data shown in all histograms represent the mean value ± SEM. *n* = 3–4 skin samples per mouse, *n* = 3–4 mice. **P* < 0.05; ***P* < 0.01; ****P* < 0.001; *****P* < 0.0001. (one-way ANOVA, Tukey's test). A, Anagen; C, Catagen; T, Telogen; Te, early Telogen; Tm, mid-Telogen; Tl, late Telogen.

© The Authors

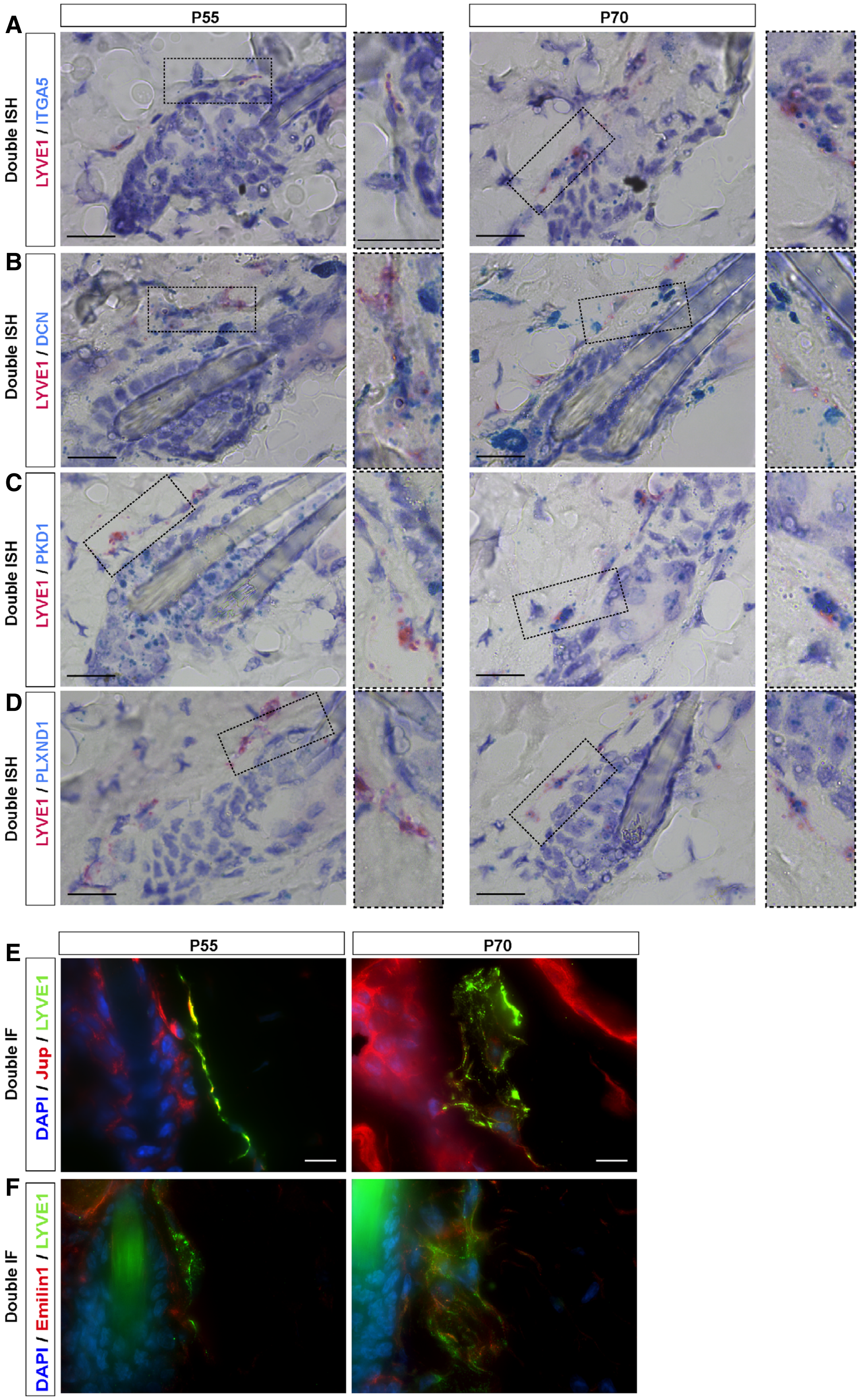

Figure EV3. Validation of RNA-seq data by double *in situ* hybridization (ISH) and immunofluorescence intensity.

A–D Representative images of RNA ISH analyses by RNAscope for *ITGA5* (A), *DCN* (B), *PKD1* (C), and *PLXND1* (D) (blue), showing the number of mRNA spots present in *LYVE1*$^+$ cells (red) in the vicinity of HF in P55 and P70 mouse back skin. Scale bar, 20 μm, insets are shown to the right as higher magnifications.

E, F Representatives images of fluorescence analyses for Jup (E) and Emilin1 (F) (red) in LYVE1$^+$ cells (green), and counterstained with DAPI (blue), in the vicinity of HF in P55 and P70 mouse skin. Scale bar, 10 μm.

 © The Authors

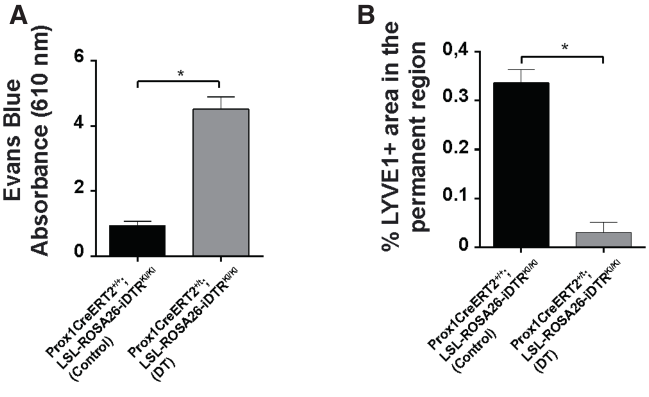

Figure EV4. Characterization of the efficiency of LV depletion in the back skin of Prox1CreERT2$^{+/T}$; LSL-ROSA26-iDTR$^{lox/lox}$ mice upon treatment with diphtheria toxin.

A Histogram of the concentration of Evans Blue (absorbance 610 nm) in the skin of Prox1CreERT2$^{+/+}$; LSL-ROSA26-iDTR$^{lox/lox}$ mice (Control) and Prox1CreERT2$^{+/T}$; LSL-ROSA26-iDTR$^{lox/lox}$ mice treated intradermally with DT. n = 3–4 skin samples per mouse, n = 3–4 mice. Data represent the mean value ± SEM. *$P < 0.05$ (unpaired Student's t-test).

B Histogram of the percentage of LYVE1^{+} area in the HF permanent region in skin sections from Prox1CreERT2$^{+/+}$; LSL-ROSA26-iDTR$^{lox/lox}$ mice (Control) and Prox1CreERT2$^{+/T}$; LSL-ROSA26-iDTR$^{lox/lox}$ mice treated intradermally with DT. n = 3–4 skin samples per mouse, n = 3–4 mice. Data represent the mean value ± SEM. *$P < 0.05$ (unpaired Student's t-test).

Figure EV5. LV depletion during the physiological HF Telogen does not induce changes in HF organization or growth, while in Anagen induces HF collapse and the loss of HF differentiation markers.

A, B Immunofluorescence of LYVE1 (green) (A) and histogram of the percentage of the LYVE1^{+} area in the permanent region (B) in adult back skin sections from Prox1CreERT2$^{+/+}$; LSL-ROSA26-iDTR$^{lox/lox}$ mice injected with vehicle (Control) and Prox1CreERT2$^{+/T}$; LSL-ROSA26-iDTR$^{lox/lox}$ mice treated with tamoxifen and intradermal DT, starting from Anagen (P49) and analyzed at the end of the treatments (P55). Scale bar, 10 μm. n = 3–4 skin samples per mouse, n = 3–4 mice. Data represent the mean value ± SEM. *$P < 0.05$ (unpaired Student's t-test).

C–F Immunofluorescence of LYVE1 (red) (C); Gata3 (green) & K6 (red) (D); AE15 (green) & K6 (red) (E); and P-cadherin (green) & K6 (red) (F) in adult back skin sections from Prox1CreERT2$^{+/+}$; LSL-ROSA26-iDTR$^{KI/KI}$ mice injected with vehicle (Control), and Prox1CreERT2$^{+/T}$; LSL-ROSA26-iDTR$^{KI/KI}$ mice treated with tamoxifen and intradermal DT, starting from Anagen (P30) and analyzed at the end of the treatments (P37). Scale bar, 50 μm. n = 3–4 skin samples per mouse, n = 3–4 mice.

▶

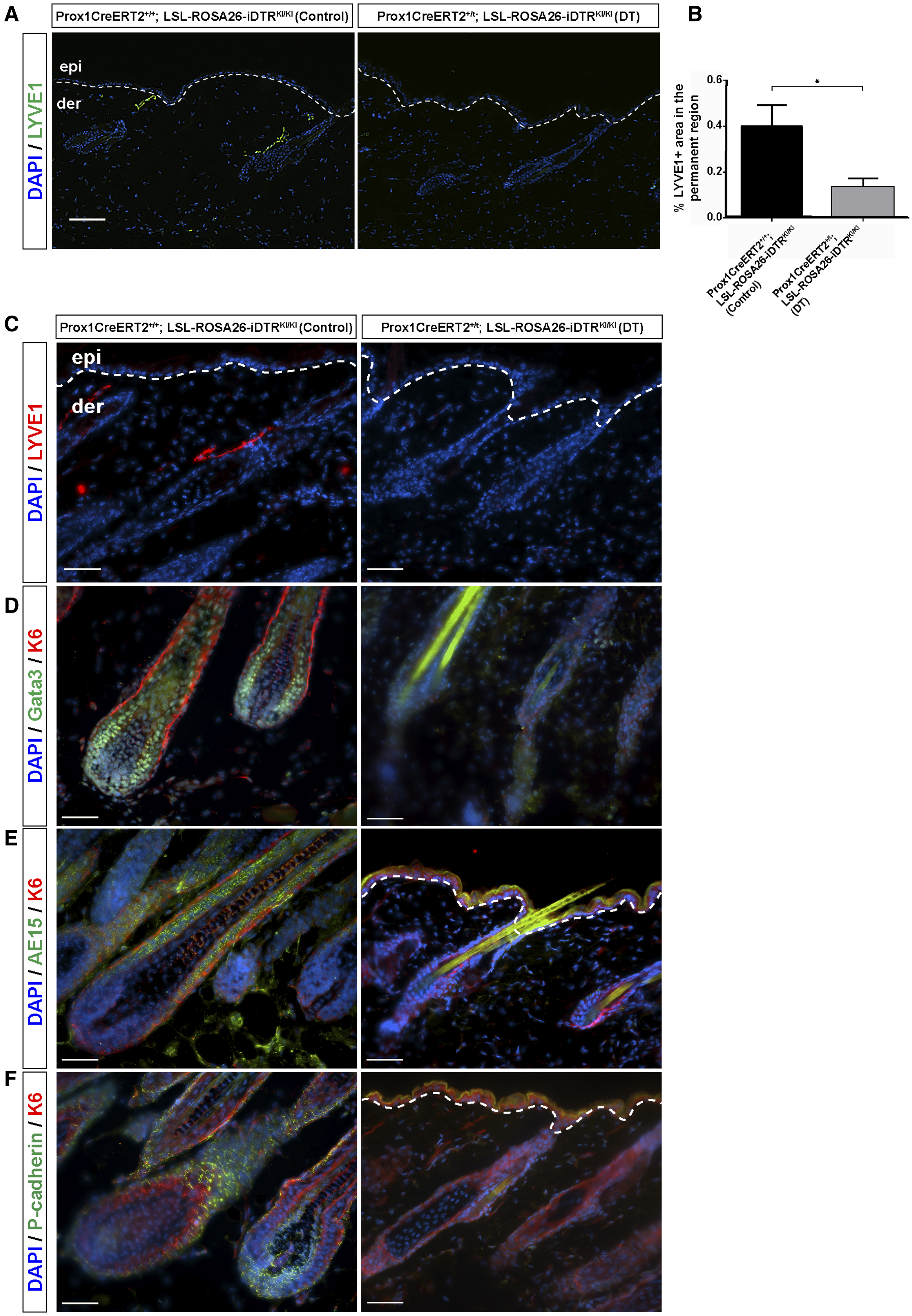

Figure EV5.

 © The Authors
