# Supplementary material for: Lymphatic vessels interact dynamically with the hair follicle stem cell niche during skin regeneration in vivo
Source: EMBO J. 2019 Sep 2;38(19):e101688. doi: 10.15252/embj.2019101688 (PMC6769427; doi:10.15252/embj.2019101688)
Supplement: Supplementary file 3 — Movie EV1 [file EMBJ-38-e101688-s003.zip › Movie_EV1_legend.docx]

**Movie EV1. 3D projection of P70 mouse backskin**, using LYVE1 (green) as lymphatic endothelial marker and counterstained with DAPI (blue). 8 fps. Bar, 50 μm.
