# Supplementary material for: Lymphatic vessels interact dynamically with the hair follicle stem cell niche during skin regeneration in vivo
Source: EMBO J. 2019 Sep 2;38(19):e101688. doi: 10.15252/embj.2019101688 (PMC6769427; doi:10.15252/embj.2019101688)
Supplement: Supplementary file 4 — Movie EV2 [file EMBJ-38-e101688-s004.zip › Movie_EV2_legend.docx]

**Movie EV2. 3D projection of whole mount immunofluorescence analyses of P70 mouse backskin,** using LYVE1 (red) as lymphatic endothelial marker and counterstained with DAPI (blue). 24 fps. Bar, 50 μm.
