# Supplementary material for: Lymphatic vessels interact dynamically with the hair follicle stem cell niche during skin regeneration in vivo
Source: EMBO J. 2019 Sep 2;38(19):e101688. doi: 10.15252/embj.2019101688 (PMC6769427; doi:10.15252/embj.2019101688)
Supplement: Supplementary file 5 — Movie EV3 [file EMBJ-38-e101688-s005.zip › Movie_EV3_legend.docx]

**Movie EV3. Intravital microscopy in the backskin of the Prox1CreERT2; Rosa-LSL-eYPF mice**. LV flow through triads of HF and across aligned HF rows in the backskin. 8 fps. n= 3 - 4 mice. Bar, 50 μm.
